# Current Status of CTCs as Liquid Biopsy in Lung Cancer and Future Directions

**DOI:** 10.3389/fonc.2015.00209

**Published:** 2015-09-30

**Authors:** Zhuo Zhang, Nithya Ramnath, Sunitha Nagrath

**Affiliations:** ^1^Department of Chemical Engineering, University of Michigan, Ann Arbor, MI, USA; ^2^Department of Internal Medicine, University of Michigan, Ann Arbor, MI, USA; ^3^Veterans Administration Ann Arbor Healthcare System, Ann Arbor, MI, USA; ^4^Translational Oncology Program, University of Michigan, Ann Arbor, MI, USA

**Keywords:** circulating tumor cells, liquid biopsy, review of literature, prognostic biomarkers, non-small-cell lung cancer, small-cell lung cancer, lung cancer

## Abstract

Circulating tumor cells (CTCs) have garnered a lot of attention in the past few decades. Isolation of these rare cells from the billions of blood cells has been a challenge until recent times. With the advent of new sensitive technologies that permit live cell isolation and downstream genomic analysis, the existing paradigm of CTC research has evolved to explore clinical utility of these cells. CTCs have been identified as prognostic and pharmacodynamic biomarkers in many solid tumors, including lung cancer. As a means of liquid biopsy, CTCs could play a major role in the development of personalized medicine and targeted therapies. This review discusses the state of various isolation strategies, cell separation techniques and key studies that illustrate the application of liquid biopsy to lung cancer.

## Introduction

Lung cancer is the leading cause of cancer worldwide, accounting for 160,000 deaths in the United States in 2014 (1) with a 5-year survival rate of 20% (2). Approximately 224,000 new cases of lung cancer were reported in 2014 (2). Smoking is the leading risk factor (3). Non-small-cell lung cancer (NSCLC) constitutes 80% of all new lung cancer cases (4). Over 50% of patients are diagnosed initially with locally advanced or metastatic disease with worse outcomes. Survival is improved through screening, early diagnosis, and treatment (5). However, currently approved screening strategies involving low-dose CT scans have a low sensitivity and high false positive rates of >90% (5). There is an unmet need for additional biomarkers that can improve the sensitivity of low-dose CT screening, particularly in patients with indeterminate pulmonary nodules. Following a diagnosis of lung cancer and stage-specific therapy, currently, the only available techniques to monitor disease progression other than clinical symptoms are periodic CT scans done every 3–6 months. Earlier detection of recurrence in cases of earlier stages of lung cancer (stages I and II) is needed to direct certain subsets of patients for treatment of oligometastatic disease; this may include surgery and/or radiation therapy. In cases of patients with more advanced lung cancers, surveillance CT scans may demonstrate progression of disease; repeat biopsies of these recurrent/progressive lesions allow us to determine the underlying resistance mechanisms, in cases of lung cancers associated with specific molecular targets (6). This type of surveillance may miss early recurrence/resistance and identification of treatable oligometastatic disease. In addition, repeat biopsies are invasive and not without risk. There is an unmet need for earlier detection of resistance in this subgroup of patients using a minimally invasive approach, which could potentially serve as decision aid for subsequent alternative therapies targeting secondary mutations or alternative pathway activation (7, 8). Furthermore, a better non-invasive approach for serial monitoring is necessary to address other clinical and research unmet needs, not only for early response assessment for targeted therapies but also for novel immunotherapies where radiologic response may lag behind or be erroneous (e.g., pseudo-progression).

**Figure 1:**
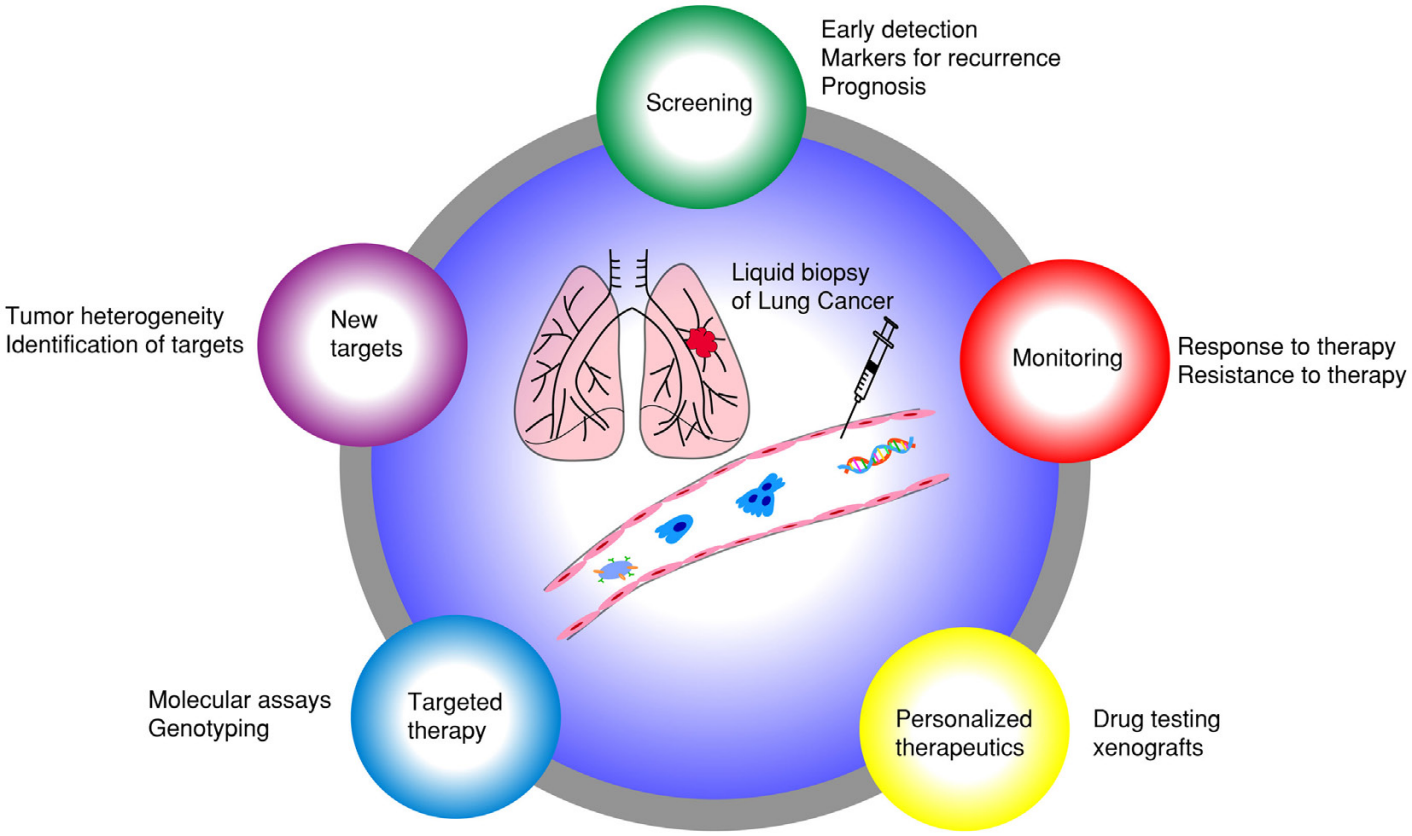
**Liquid biopsy of lung cancer: different applications of using CTCs as surrogate biomarkers in lung cancer**.

**Figure 2:**
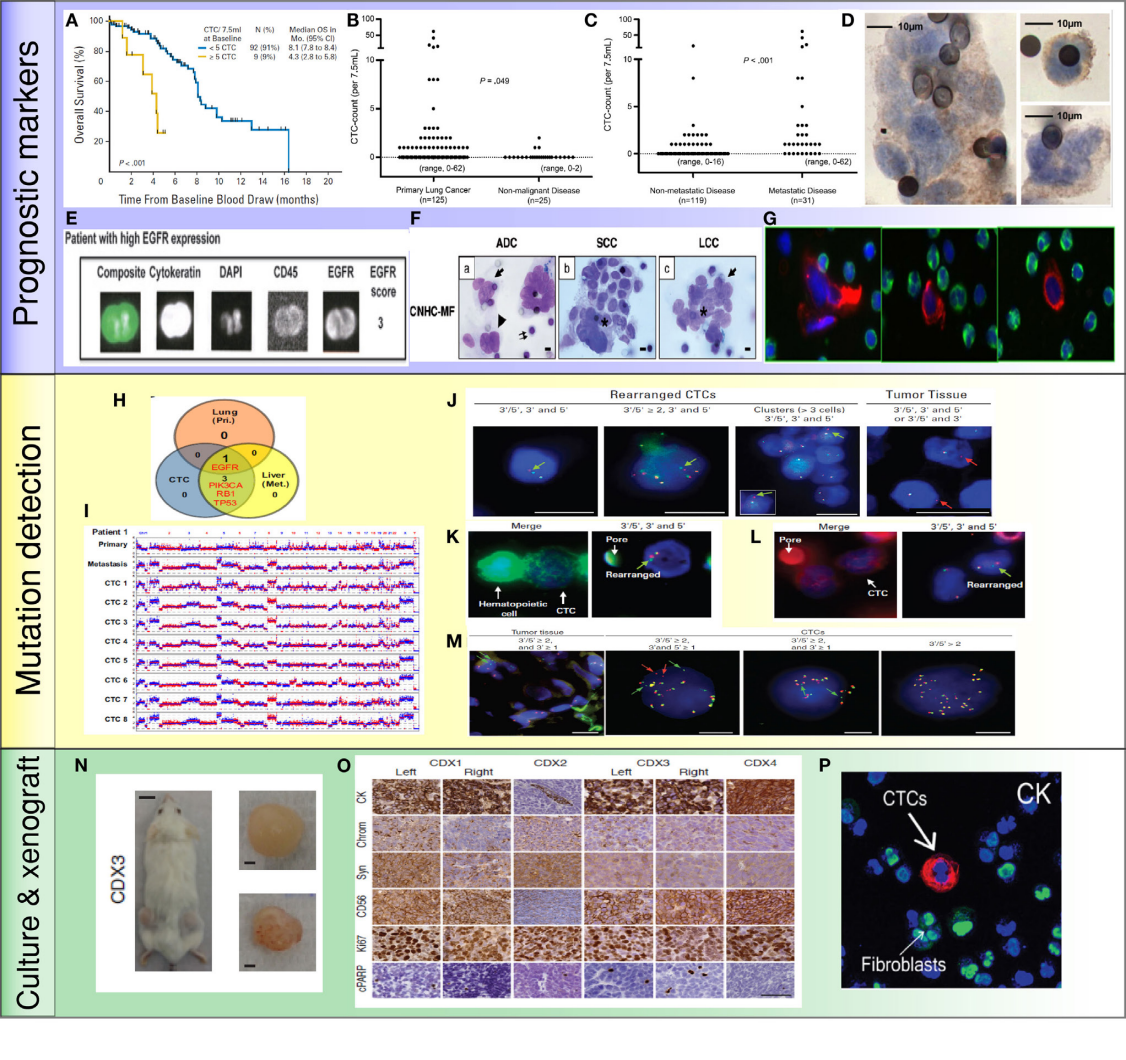
**Application of CTCs in lung cancer: (A) less than 5 CTCs/7.5 ml of blood predicted improved survival by CellSearch system** (19). **(B,C)** Higher numbers of CTCs were detected in metastatic lung cancer than cancer without distant metastasis (35). **(D)** NSCLC CTCs were detected by ISET technology and stained positive for EGFR (36). **(E)** NSCLC CTCs were isolated by CellSearch system and stained positive for EGFR and CK (33). **(F)** Morphologic features of CTCs from different histologies of NSCLC (37). **(G)** CTCs were detected by HD-CTC assay and stained positive for CK (red) and negative to CD45 (green) (38). **(H,I)** Mutations were detected in CTCs, primary tumors, and metastatic sites. Copy number variation patterns among single CTCs, primary tumor, and metastatic sites (39). **(J)** ALK rearrangement patterns in CTCs and primary tumor (40). **(K,L)** ALK rearranged CTCs stained positive for vimentin **(K)** and N-Cadherin **(L)** (40). **(M)** ROS1-rearranged CTCs were compared to primary tumor (41). **(N)** CTCs isolated from SCLC patients generated tumor in a mouse (42). **(O)** CTC-derived xenografts were stained for different protein markers (42). **(P)** NSCLC CTCs were isolated and expanded by a microfluidic co-culture model and stained positive for CK (27).

Emerging research in blood-based biomarkers provide new opportunities to diagnose lung cancer earlier and also to assist with detection of earlier recurrence. These biomarkers include circulating tumor cells (CTCs) (9) and circulating cell-free nucleic acids (CfNA) (10–12). CfNA are released from apoptotic or necrotic tumor cells (13). Besides plasma, tumor nucleic acids have also been detected in other fractions of blood, such as platelets (14), extracellular vesicle exosomes (15), and buffy coat (leukocyte-enriched) (16). New technologies are being developed to increase the sensitivity and specificity of cfNA detection in the blood (17). CTCs, on the other hand, have been extensively studied as prognostic and pharmacodynamic biomarkers in many cancers (18–22). Both cfNA and CTCs were demonstrated to correlate with tumor burden and revealed genetic signatures of primary and metastatic tumors (23, 24). CTC processing technologies, however, have unique advantages over cfNA, by permitting a vast array of molecular and functional studies, including cell culture, xenograft implantation, and *ex vivo* drug testing (Figure 1) (25). CTCs represent a subset of tumor cells that have acquired the ability to disseminate from the primary tumor and intravasate to the circulatory system (26). Sampling CTCs may be a viable non-invasive alternative to tissue biopsies for diagnosis of lung cancers. In many patients, however, CTCs are quite low in number, and need to be isolated from an overwhelming majority of blood cells (1 CTC: 1 billion blood cells). We have reported capability of detecting and characterizing CTCs from early stages of lung cancer (27). CTCs have demonstrated utility in surveillance of patients and their changing numbers predict progression-free survival (PFS) and overall survival (OS) in several cancers (19, 28). Additionally, CTCs have been proposed as surrogate biomarkers in a multitude of research areas, including the selection of neoadjuvant and adjuvant therapy, detection of recurrent disease, and as pharmacodynamic biomarkers of novel therapeutics (22, 26, 29–34). In this review, we summarize current technological and scientific advancements in CTC research specifically pertaining to lung cancer and discuss possible future directions (Figure 2).

## CTC Isolation Technologies

Circulating tumor cells have now been proposed as surrogate biomarkers in over 270 clinical trials (10). However, to date, CTCs have not been incorporated into routine clinical practice for management of patients with cancer. The efforts to identify biological relevance and clinical utility of CTCs parallel the development of CTC isolation technologies. There are several key parameters worthy of consideration when designing a method to isolate CTCs: (a) specificity, (b) sensitivity, (c) purity, (d) viability, and (e) throughput. All the downstream assays, such as molecular and genomic analysis and culturing for *ex vivo* drug testing, depend on these factors. We will discuss about pros and cons associated with current isolation technologies in general and specifically as they pertain to lung cancer (Table 1).

**Table 1:** **Comparison of CTC isolation technologies**.

Technology	Approach	Flow rate (ml/h)	Recovery cell lines	Purity WBCs (ml)	Patient samples	Whole blood	Genomic analysis	Live cells	Culture	Drug testing
CellSearch	EpCAM-coated magnetic beads	NA	>80%	Low	<50% in breast (43) 32% in lung cancer, 5CTCs/7.5ml (19)	N	N	N	N	N
Epic Sciences	No enrichment, RBCs lysed blood deposited on slides	NA	NA	None	73% in lung cancer (38), 55% in melanoma (112)	N	Y, single cell for copy number	N	N	N
Mag Sweeper	Flow through immunomagnetic capture		62 ± 7%		100% in metastatic breast cancer, 12CEpCs/9 ml (113)	Y, need dilution	Y	Y	N	NA
ISET	Size-based filtration	NA	One CTC per 1 ml of blood	NA	80% in lung cancer (36, 40, 68)	N	Y, FISH	N	N	N
CTC iChip	Size-based separation +ve or −ve selection with mag beads	9.6	>95% for −ve 78–98% for +ve	>10,000 for −ve <10,000 for +ve	90% from multiple types of metastatic cancers, including lung cancer (64)	Y, not a single step	Y, single-cell RNA expression	Y	Y	Y
FACS Sorting	Surface marker-based selection	Very low	NA	Very Low	<10% (99)	Y	Y	Y	Y	N
RosetteSep kit	Depletion of WBCs	NA	NA	NA	NA (42, 101, 109)	Multiple steps	Y	Y	Y	NA
CTC chip	Positive selection	1	>95%	NA	72% in lung cancer (27)	Y	Y	Y	Y	N
GO Chip	Nanopillars with graphene oxide	1–3	>95% 2–5 CTCs	<1,000	>95% sensitivity, 10 CTCs/ml (46)	Y	Y	Y	Y	N

Collectively, there are two major approaches; one is anti-epithelial cell adhesion molecule (EpCAM) dependent while the other is EpCAM independent. The FDA approved CellSearch technology utilizes EpCAM-coated magnetic beads to isolate CTCs in a multitude of cancers in spite of limited detection efficiency (32% in lung cancer) (19, 43–45). Microfluidic-based technologies have changed the existing paradigm for recovery of CTCs. Microfluidic chips coated with EpCAM and microfluidic systems utilizing immunomagnetic principles have been shown to capture CTCs from lung cancer samples with 100% efficiency (46–49). These antibody-based microfluidic devices have the advantage of high sensitivity, low numbers of white blood cells contamination (can be as low as 1,500 WBCs), as well as preserving the viability of CTCs due to minimal handling of whole blood. The drawback is that they suffer from limited throughput due to low flow rates (1–3 ml/h) and a requirement for antibody–antigen interaction. Another problem with EpCAM-dependent methods is that they can only capture a subset of CTCs and miss cells undergoing epithelial–mesenchymal transition (EMT) (10). Wit et al. recovered lung CTCs by filtration from the waste of CellSearch system (50). The percentage of patients having more than 5 cells per 7.5 ml of blood increased from 15% (EpCAM positive) to 41% (EpCAM positive and negative). This suggested that including the EpCAM negative population increased CTC recovery.

By contrast, the label-free approaches to isolate CTCs do not rely on the expression of specific cell surface markers but instead on inherent CTC properties such as size, deformability, or dielectric susceptibility, and/or negative selection of WBCs (51–60). While improvements in size-based and other physical separation techniques have allowed higher throughput over the years, they suffer from limitations related to heterogeneity of tumor cells, contamination with blood cells and result in lower yield and specificity compared with the antigen-based systems (61). For example, CTCs within a patient may have a wide range of sizes (>4–30 μm) and many of them may overlap in size with blood cells (62). More recently, several new integrated platforms have emerged for CTC isolation. Liu et al. introduced an integrated device that separates blood cells and CTCs by deterministic lateral displacement, followed by an affinity-based enrichment (9.6 ml/h) (63). The CTC-iChip by Ozkumur et al. combines magnetic labeling and high throughput sorting of cells (8 ml/h) (64), which is based on the principle of conjugating capturing antibodies on magnetic particles and enriching rare cells by applying external magnetic forces (65). While EpCAM-independent systems allow high throughput and an unbiased surface marker-independent approach that can capture cells undergoing EMT (66), the need for multiplexing and pre-processing of blood samples make it cumbersome and time consuming. Chang et al. employed similar principles by labeling CTCs with antibody cocktail conjugated with magnetic beads followed by size-based filtration to trap CTCs on chip for immunofluorescence staining (67). This system also operates at high flow rates (2 ml/min) but requires RBC lysis and the average WBCs contamination was around 4000.

Other label-free technologies that are not microfluidic based are also employed in clinical evaluation of lung CTCs. The isolation by size of epithelial tumor cells (ISET) technology, isolating CTCs based on their larger size, is among the earliest developed EpCAM-independent approaches which filter CTCs from blood cells as they pass through a membrane filter (68). CTCs were detected in 80% of samples from stages IIIA–IV NSCLC patients using ISET compared with 23% using CellSearch (36). Using the same approach, CTCs were present in 65% of NSCLC patients in a more recent report (69). In another study, an automatic microscope scanning and analysis technology called high-definition CTC (HD-CTC) assay was utilized to examine CTCs from stages I–IV NSCLC patients (38, 70). This technology permits high-resolution imaging of CTCs and is not biased toward size or surface markers. Recently, DNA aptamers were utilized to isolate CTCs from NSCLC patients (71). CTCs were identified in 86% of the samples that were positive for aptamers and pan-CK. The ISET, HD-CTC assay, and aptamer approach require RBC lysis and have limited purity of isolated CTCs, therefore posing constraints on molecular and functional studies of the cells.

In summary, CTC technologies have evolved rapidly in the last decade, yet there is none that has FDA approval other than CellSearch. However, to incorporate CTCs into basic as well as small cohort clinical research, there are more tools than ever before, with microfluidic devices leading the way with higher sensitivity. Any ideal CTC technology should offer high throughput, minimal handling (whole blood) that can separate live CTCs with high sensitivity and specificity. Presently, there is no single technology that is optimal for every downstream analysis; the choice of technology is driven more by the end user application and ease of accessibility to the technology. Immunoaffinity-based technologies offer both sensitivity and specificity albeit with dependence on the known biomarker. A high throughput system that requires minimal pre-manipulation of whole blood and that can operate with either positive selection or negative depletion approach seems to be most promising for lung cancer CTC isolation. Furthermore, the efficiency of positive selection depends on the discovery of lung cancer-specific surface markers such that a cocktail of capturing antibodies can be applied to target a broader range of lung CTCs.

## CTCs as Prognostic and Predictive Markers in Lung Cancer

Previously, the oncology community believed that there was little merit in diagnosing recurrence or progression earlier in patients who had surgery for earlier stages of lung cancer or following initial therapy for locally advanced/metastatic NSCLC. This was related to poor therapy choices at recurrence/progression that often does not alter clinically significant outcomes such as PFS and OS. There has been a rethink of this approach in a small, but significant minority of patients. This relates to the emerging field of therapy directed at oligometastatic disease such as local radiation or use of immunotherapy or newer biologics that may render patient disease free for a significant amount of time, even if OS is not affected. Many of these therapies are also better tolerated with broader therapeutic windows. We will, in this section, outline various studies relating CTCs to prognosis in lung cancer as well as studies that predict therapy response.

Hofman et al. used ISET technology to isolate CTCs from 208 NSCLC patients with stages I–IV cancer. Fifty percent of these patients had CTCs by morphological examination (37). A cut-off value of >50 corresponded to shorter PFS and OS. There was, however, no direct correlation between numbers of CTCs and disease stage, or other clinicopathologic parameters. Therefore, CTCs and tumor staging appeared to be independent prognostic factors. In another study using the CellSearch system, there were greater numbers of CTCs in metastatic lung cancer patients (*P* < 0.001) compared to patients without distant metastases (35). Similarly, another study using the CellSearch system found that in 101 patients with stage III/IV NSCLC, numbers of CTCs were higher in stage IV compared to stage III patients (19). With a threshold of 5 CTCs in 7.5 ml blood, patients were categorized into favorable and unfavorable groups. Both the PFS (6.8 vs. 2.4 months) and OS (8.1 vs. 4.3 months) were higher in the favorable group than the unfavorable (*P* < 0.001). Additionally, CTC numbers decreased with one cycle of chemotherapy. Reduction in numbers of CTCs with therapy correlated with improved PFS (6.9 vs. 2.4 months; *P* = 0.005) and OS (8.8 vs. 3.9 months; *P* < 0.001). This study highlighted that CTC numbers were not only prognostic, but also that a change in CTC number with therapy predicted disease progression dynamically.

Dorsey et al. investigated the change of CTC number in patients with localized NSCLC undergoing radiation treatment. Using a telomerase-based detection assay, 65% of the patients were positive for CTCs prior to treatment. CTC numbers significantly reduced after radiation (9.1 vs. 0.6 CTCs/ml; *P* < 0.001). This study suggested that analyzing CTC can serve as “real-time liquid biopsies” accompanying treatment to monitor tumor progression (72). Several studies examining CTCs in advanced NSCLC patients receiving chemotherapy have shown that >2 CTCs/7.5 ml or any increase in CTC numbers after therapy predicted lower OS and PFS (*P* = 0.05) (33, 34, 73). To improve detection sensitivity, CTCs from pulmonary vein blood were examined in patients undergoing surgery (74–76). Compared to peripheral blood CTCs (2 out of 30 positives), pulmonary CTCs were present in 22 out of 30 samples before surgery (0–1122 cells/2.5 ml, median, 4 cells/2.5 ml) (75). Surprisingly, the number of pulmonary CTCs increased significantly after surgical manipulation (0–1855 cells/2.5 ml, median, 60 cells/2.5 ml); this increase also correlated with pathological evidence of microscopic lymphatic invasion (*P* = 0.043). Chudasama et al. investigated the effect of endobronchial cryotherapy (EC) on shedding of CTCs before and after the procedure in peripheral blood (77). CTC count increased following cryotherapy in 15 out of 20 advanced stage patients (*P* = 0.0086) which predicted poor prognosis during follow-up. In summary, these studies suggest that monitoring change of CTC numbers during therapy is prognostic for NSCLC. An increase of CTC counts may entail additional follow-up examinations.

More recently, several groups have reported on the prognostic value of CTC clusters called circulating tumor microemboli (CTM) (36, 70). Krebs et al. observed the prevalence of CTM by ISET technology in 43% of patients with stage IIIB/IV NSCLC (36). In another study using HD-CTC assay, 50% of NSCLC patients with stages I–IV disease had CTM (70). It was shown that CTM can be used to diagnose lung cancer when combined with clinical and imaging data. The existence of CTMs was also observed in pulmonary venous (PV) blood of patients with NSCLC (76). Among 130 patients tested, 74% of them were positive for CTCs. CTMs were detected in 33% of samples which predicted tumor recurrence and worse disease-free survival rate (*P* < 0.01).

Other studies correlated prognosis to the presence/absence of protein expression of CTCs in NSCLC. As demonstrated by Wu et al., CTCs in multiple types of cancer, including lung cancer, harbored a mixed population of epithelial and mesenchymal phenotypes (78). Nel et al. stained CTCs for both epithelial markers such as EpCAM and pan-cytokeratin (CK) as well as mesenchymal markers such as N-cadherin and CD133 (79). Different subsets of CTC populations were identified with heterogeneous combinations of epithelial and mesenchymal characteristics. CD133 expression correlated positively with N-cadherin. The presence of these mesenchymal markers predicted shorter PFS (2 vs. 8 months, *P* = 0.003) likely due to emergence of chemoresistant populations.

Small-cell lung cancer (SCLC) accounts for 13% of newly diagnosed lung cancer and is considered aggressive with early dissemination and poor prognosis (80). Hou et al. demonstrated that CTCs were present in 85% of SCLC compared to 21% in NSCLC patients (19, 81). Higher CTC numbers were noted in SCLC than NSCLC; >50 CTCs/7.5 ml of blood predicted shorter PFS (4.6 vs. 8.8 months; 95% CI) and OS (5.4 vs. 11.5 months; 95% CI). A reduction in CTC number after chemotherapy was associated with longer PFS (9.6 vs. 4.1 months; 95% CI) and OS (10.4 vs. 4.1 months; 95% CI). Huang et al. evaluated prognostic significance of CTCs in SCLC. CTCs were enumerated before and after chemotherapy (82). A reduction of CTCs was observed in 16/26 patients after treatment. However, CTC count at baseline and the percentage change of CTCs were not statistically significantly associated with survival. A summary of the studies investigating the prognostic value of CTCs in lung cancer is shown in Table 2.

**Table 2:** **CTCs as prognostic markers in lung cancer**.

Study	Technology	Sensitivity (% of patients positive for CTCs)	Prognostic significance
Hofman et al. (37) 208 NSCLC patients (stages I–IV)	ISET	50%	>50 CTCs corresponded with shorter OS and PFS
Tanaka et al. (35) 125 lung cancer patients (stages I–IV) 25 patients with non-malignant diseases	CellSearch	30% in all patients 71% in metastatic patients	CTC count was higher in lung cancer than non-malignant patients. CTC count was higher in patient with distant metastasis
Kreb et al. (19) 101 NSCLC patients (Stages III–IV)	CellSearch	21% at baseline (32% at stage IV, 7% at stage IIIB)	>5 CTCs/7.5 ml blood predicted shorter PFS and OS. A reduction in CTC count after chemotherapy predicted improved survival
Dorsey et al. (72) 30 NSCLC patients received radiation therapy	Telomerase-based assay	65% before RT	CTC count decreased in patients responding to RT
Juan et al. (73) 37 NSCLC patients [Advanced stage (IIIB–IV)]	CellSearch	24% at baseline	No significant prognostic conclusion was made
Muinelo-Romay et al. (34) 43 NSCLC patients (stages IIIB and IV)	CellSearch	42% at baseline	>5 CTCs/7.5 ml blood at baseline predicted shorter PFS and OS. CTC count increase during chemotherapy correlated with worse PFS and OS
Punnoose et al. (33) 41 NSCLC patients (Advanced stage)	CellSearch	76% at baseline	Reduction in CTC count after chemotherapy predicted longer PFS
Sienel et al. (74) 62 NSCLC patients (stages I–III)	Ficoll-Hypaque Centrifugation	18% in pulmonary venous (PV) blood	Presence of CTCs in PV blood was associated with shorter survival especially in patients with lymph node involvement
Hashimoto et al. (75) 30 NSCLC patients (stages I–IV)	CellSearch	73% in PV blood before surgery	CTC count in PV blood significantly increased after surgery, which predicted lymphatic tumor invasion
Funaki et al. (76) 130 NSCLC patients (stages I–IV)	RosetteSep kit	74% in PV blood after tumor resection Circulating tumor microemboli (CTM) in 33%	The presence of CTM in PV blood predicted worse PFS
Chudasama et al.(77) 20 NSCLC patients (stages III–IV)	ScreenCell	25% at baseline, 75% after endobronchial cryotherapy (EC)	CTC count increased after EC
Carlsson et al. (70) 104 NSCLC patients (stages I–IV) 25 patients with benign diseases	HD-CTC assay	50% positive to CTM	CTM along with clinical and imaging data can serve as predictor of malignant vs benign diseases
Pirozzi et al. (114) 45 NSCLC patients (stages I–III)	Ficoll-Hypaque Centrifugation	24% in PV blood	No association found between presence of CTCs and prognosis
Nel et al. (79) 43 NSCLC patients (stages IIB–IV)	Ficoll-Paque CD45 magnetic depletion	100%	Presence of mesenchymal markers CD133 and N-cadherin in CTCs predicated shorter PFS
Hou et al. (81) 97 SCLC patients	CellSearch and ISET	CTCs in 85% CTM in 32%	More than 50 CTCs/7.5 ml blood predicated shorter OS
Huang et al. (82) 26 SCLC patients	CellSearch	Not reported Median CTC count at baseline is 75 (0–3430)	CTC count decreased after chemotherapy CTC count at baseline and change of CTC numbers after treatment not associated with survival

Taken together, several studies have demonstrated the prognostic utility of CTCs in lung cancer. CTC count and change of CTC number after surgery, radiation, and chemotherapy may serve as predictors of recurrence. At the current time, however, CTCs are not routinely used as prognostic or predictive markers in clinics. There are several reasons for this. Most of the previous studies used CellSearch or traditional approaches without pre-enrichment, which limited sensitivity of the tests in detecting CTCs. Many of the studies had small sample sizes (<100) limiting statistical significance. This resulted in contradictory or inconclusive findings. Given newer and extremely sensitive technologies that allow isolation and accurate characterization of CTCs, large numbers of patients within specific stages of lung cancer need to be enrolled. The stringent biomarker studies need to use training and test sets that will allow independent validation and reproducibility.

## Applications of CTCs in the Era of Targeted Therapies in Lung Cancer

The past two decades have seen a large discovery effort such that lung cancer is not considered one homogeneous cancer. Over 64% of all lung cancers have an underlying driver mutation that is responsible for proliferation of the cancer and many of these mutations are mutually exclusive (83). Nearly 30% cases of these driver mutant lung cancers have an approved therapy (targeted therapy). The most common ones are adenocarcinomas (AC) that are associated with mutations in the *EGFR* gene or rearrangements in the *ALK* and *ROS-1* gene (84). Additional genomic aberrations include those in *BRAF, AKT1, ERRB2, PIK3CA*, and fusions in *RET* (85). Detection of mutation by biopsy may not fully reflect intratumoral heterogeneity (86). In this regard, sampling CTCs as “liquid biopsy” may complement solid biopsy to inform effective targeted therapies. Liquid biopsy is also non-invasive allowing dynamic monitoring of disease progression (13).

One of the earliest investigations was identifying *EGFR* mutations in CTCs from metastatic NSCLC known to harbor these mutations. In 11 out of 12 patients, expected mutations were validated, including the appearance of the resistance mutation T790M. In this study, CTC numbers paralleled radiographic response and offered first insights into genomic profiling of CTCs as a way to monitor genotypic changes during therapy (24). Two recent studies examined *EGFR* mutation in advanced NSCLC patients. Marchetti et al. demonstrated that *EGFR* mutation was detected in CTCs of 84% of the patients carrying *EGFR*-mutant primary tumors (87). In 94% of the cases, mutations found in CTCs matched the mutations in tumor tissues. The unmatched mutations in CTCs and primary tumors were likely due to tumor heterogeneity between primary lesions and metastatic sites. Breitenbuecher et al. utilized a RT-PCR assay to detect in-frame deletions in the *EGFR* exon 19 (88). All eight *EGFR*-mutant patients demonstrated identical mutations in the CTCs. *EGFR* mutations were also detected from circulating DNA of advanced lung AC patients with 73% sensitivity (89). Both CTCs and cfDNA can be used in future research to determine the “best in class” *EGFR* tyrosine kinase inhibitors (TKI) for individual patients.

Other studies focused on investigating *ALK* rearrangement in CTCs (40, 90). By performing filter-adapted fluorescent *in situ* hybridization (FA-FISH), researchers identified unique *ALK*-rearranged pattern in CTCs with a mesenchymal phenotype. This unique population of CTCs may be highly invasive, behaving as metastasis initiation cells (91). Adapting the similar approach, *ROS1* rearrangement was investigated in NSCLC CTCs and compared to tumor biopsy specimens (41). Among four patients tested, CTCs harbored similar split patterns as tumors but exhibited an increase in *ROS1* copy number. The number of *ROS1*-rearranged CTCs increased in one patient who did not respond to crizotinib treatment. In another study, whole-genome amplification of single CTCs from lung cancer patients was performed followed by analyzing copy number variation (CNV) in addition to somatic mutations (39). It was demonstrated that CTCs obtained from the same patient exhibited similar CNV pattern but was distinguishable from CTCs obtained from a different histology of lung cancer. These studies suggest that profiling CTC genome can predict cancer progression as well as emergence of secondary resistant mechanisms to be further targeted by therapy.

## CTCs as Biomarkers for Early Diagnosis of Lung Cancer

Sensitive detection of CTCs provides opportunities for early diagnosis of lung cancer. CTCs can be shed by primary tumor even at early stages of tumor development (92, 93). It was demonstrated that the presence of CTCs in 5 out of 168 chronic obstructive pulmonary disease patients predicted occurrence of lung nodules 1–4 years after initial detection of CTCs (94). In one study, CTCs were isolated from 84% of lung cancer patients of various stages, including early stage of lung cancer (57.1%) (95). CTCs were identified with CD45-FISH method that was reported to increase detection sensitivity by including cells deficient in epithelial markers like CK. Two studies utilized tumor-specific ligand folate and an oligonucleotide followed by qPCR and immunofluorescence staining to identify NSCLC CTCs (96, 97). CTCs were observed in more than 70% of all stages with 67.2% in stage I cancer. It was further demonstrated that CTCs can be more sensitive for early diagnosis of lung cancer than blood serum markers such as cyfra21-1 or CEA. More recently, one study evaluated CTCs from potential lung cancer patients to predict malignancy of the lung lesions as a way to circumvent sampling bias by solid biopsy (98). CTCs isolated shared similar morphological features and histology (72%) with biopsy specimens. In stage I patients tested (42%), the numbers of CTCs correlated with tumor size (*P* = 0.001). Our group also demonstrated that CTCs are detectable in early stages of lung cancer (68%) (27). Early diagnosis of cancer aided by liquid biopsy is challenging due to low abundance of CTCs present; therefore, it is necessary to develop more sensitive and specific technologies that allow more inclusive characterization methodologies that will aid early detection of lung cancer.

## Current Applications: Culture of CTCs and Xenografts

While the exploration of innovative technologies for enhanced CTC isolation is always in the forefront of research, *ex vivo* culturing and *in vivo* xenograft models has gradually gained momentum in the field. Zhang et al. sorted a subset of breast CTCs to form cell lines and tumors in mice (99). This model helped to identify a novel gene signature associated with development of brain metastasis. Yu et al. successfully cultured breast CTCs followed by *in vivo* implantation and drug testing (100). Cayrefourcq et al. established and characterized a cell line derived from colon CTCs which served as a model for studying metastasis and testing treatment agents (101). Higher numbers of CTCs in SCLC allow *ex vivo* culture of these cells and formation of CTC-derived xenografts for drug screening and mutation detection. In the study by Hodgkinson et al., CTCs from SCLC patients were injected directly to mice (42). Samples with CTC count >400/7.5 ml successfully gave rise to tumors in mice in 2–4 months. This study demonstrated that CTCs from SCLC were tumorigenic and that CTC-derived xenografts termed CDXs mirrored the corresponding tumor biopsy specimens. CDXs can potentially serve as *in vivo* drug testing models with responses similar to those seen in patients. Next-generation sequencing (NGS) revealed similar genomic aberrations in CDXs as seen in SCLCs. Our group recently demonstrated capability of using a co-culture model to isolate and culture CTCs from patients with stages I–III NSCLC (27). A microfluidic co-culture model utilizing hydrogel and cancer-associated fibroblasts was developed to facilitate CTC culturing. This permitted histological characterizations as well as genomic comparison between CTCs and matched primary tumors. Culturing of CTCs can overcome a critical limitation related to the rarity of these cells. This will allow further *in vivo* and functional studies.

## Future Directions

Over the past decade, the advancement of technological innovation to isolate CTCs has allowed investigation of their clinical utility (102, 103). We now understand that CTCs contain heterogeneous populations of both epithelial and mesenchymal phenotypes (104). They harbor genetic alterations that correspond to primary tumors and metastatic sites. The discordant or unique mutations carried by CTCs that are absent in primary tumors reflect heterogeneity in primary tumor or small amount of subclonal populations that are missed by conventional sequencing methods (105). Together with cfDNA, CTCs have been shown as promising surrogates of tumor burden and activating mutations for targeted therapies. Specifically, CTCs offer opportunities to perform biological studies such as phenotypic and histological characterization, invasion and migration assays, *in vitro* expansion, drug testing, and use as xenografts in animal models. Despite current advances in the field, CTC markers still fall short, when it comes to factoring inter-tumor and intra-tumor heterogeneity (106). We have seen this manifests as discordance in markers between primary/metastatic cancers and CTC genotypes or phenotypes. Additionally, there is still the problem of contaminating white blood cells, despite the emergence of several technologies that enable positive selection and negative depletion of leukocytes. These factors along with a low yield in earlier stages of lung cancer handicap functional studies related to CTCs [e.g., ability to detect metastasis initiation properties of lung CTCs as demonstrated in other cancers using EPISPOT assay (101, 107) or invasion capabilities (27, 108)]. *In vivo* studies of CTCs through generating CTC-derived xenografts generally require larger numbers of cells (42, 109) which are hard to obtain from early stage cancer patients; this can be to some extent overcome by increasing blood throughput and sensitivity of isolation methods. Biology of metastasis as related to a cascade of events has vast implications in drug development. The study of CTCs opens up a window for understanding this process. One study found that the *WNT2* gene was enriched in breast CTCs and another study showed that genes involved in ECM were highly expressed in pancreatic CTCs (110, 111). These findings suggest that these genes and pathways can be targeted therapeutically to halt metastasis and likely improve survival. However, this type of study has not been done with lung cancer. Understanding metastasis initiating capabilities of CTCs from primary lung cancer will have a huge impact in providing specific adjuvant therapies targeting at these CTCs to reduce metastasis and improve survival. The next few years will allow us to further study these biological processes in depth and allow meaningful translation into the clinic.

## Conflict of Interest Statement

The authors declare that the research was conducted in the absence of any commercial or financial relationships that could be construed as a potential conflict of interest.
